# Supplementary material for: Advancing EDGE Zones to identify spatial conservation priorities of tetrapod evolutionary history
Source: Nat Commun. 2024 Sep 3;15:7672. doi: 10.1038/s41467-024-51992-5 (PMC11377708; doi:10.1038/s41467-024-51992-5)
Supplement: Supplementary file 1 — Supplementary Information [file 41467_2024_51992_MOESM1_ESM.pdf]

Supporting information

Advancing EDGE Zones: spatial priorities for the conservation of tetrapod evolutionary history

Sebastian Pipins*, Jonathan E.M. Baillie, Alex Bowmer, Laura J. Pollock, Nisha Owen¶, Rikki Gumbs[4]¶

*Corresponding author. Email: sebastianpipins@ontheedge.org

¶ These authors contributed equally to this work.

Supporting Information Contents

Supplementary Figure 1. EDGE species richness per ecoregion.

Supplementary Figure 2. Regressions of latitude against threatened evolutionary history and EDGE species richness.

Supplementary Figure 3. EDGE tetrapod species richness.

Supplementary Figure 4. Spatial congruence of EDGE tetrapod groups.

Supplementary Figure 5. Threatened evolutionary history of tetrapod groups.

Supplementary Figure 6. Comparison of species richness and threatened evolutionary history.

Supplementary Figure 7. The irreplaceability of EDGE Zone grid cells.

Supplementary Figure 8. Threatened evolutionary history prioritisation with complementarity at a resolution of 193 km x 193 km.

Supplementary Figure 9. Comparison of a branch-length vs a median EDGE score approach for the selection of priority grid cells.

Supplementary Figure 10. EDGE rarity prioritisation with complementarity.

Supplementary Figure 11. Weighted endemism prioritisation

Supplementary Figure 12. Biodiversity Hotspots.

Supplementary Figure 13. Evolutionary Distinctiveness of tetrapod species with absent range data.

Supplementary Figure 14. The complementary set of grid cells needed to capture 100% of threatened tetrapod evolutionary history.

Supplementary Note 1. A comparison of using summed EDGE scores vs phylogenetic branch lengths for the calculation of threatened evolutionary history.

Supplementary Note 2. Predictors of irreplaceability in EDGE Zone grid cells.

Supplementary Note 3. Comparison of the tetrapod threatened evolutionary history within and outside of EDGE Zones.

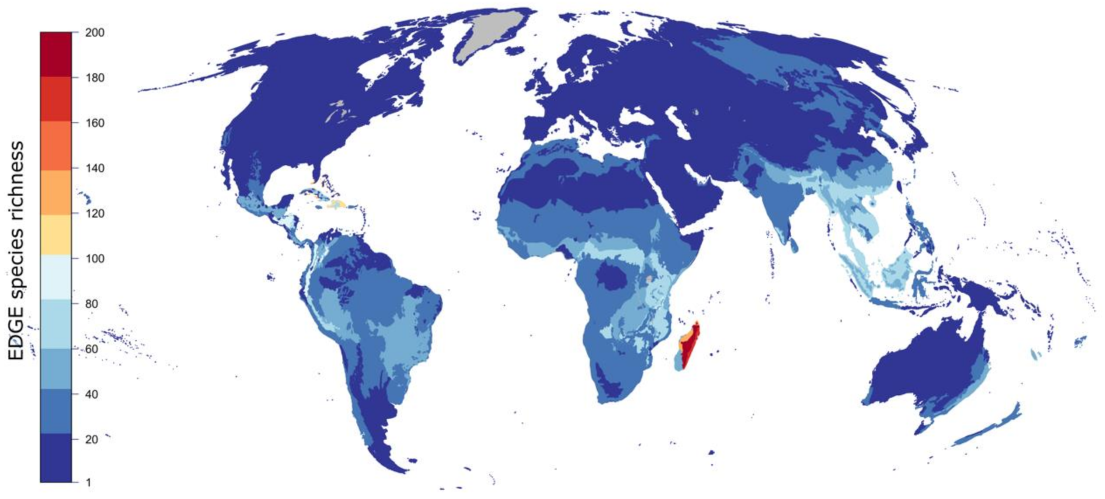

Supplementary Figure 1: EDGE species richness per ecoregion. The number of Evolutionarily Distinct and Globally Endangered (EDGE) species found in each ecoregion. Source data are provided as a Source Data file.

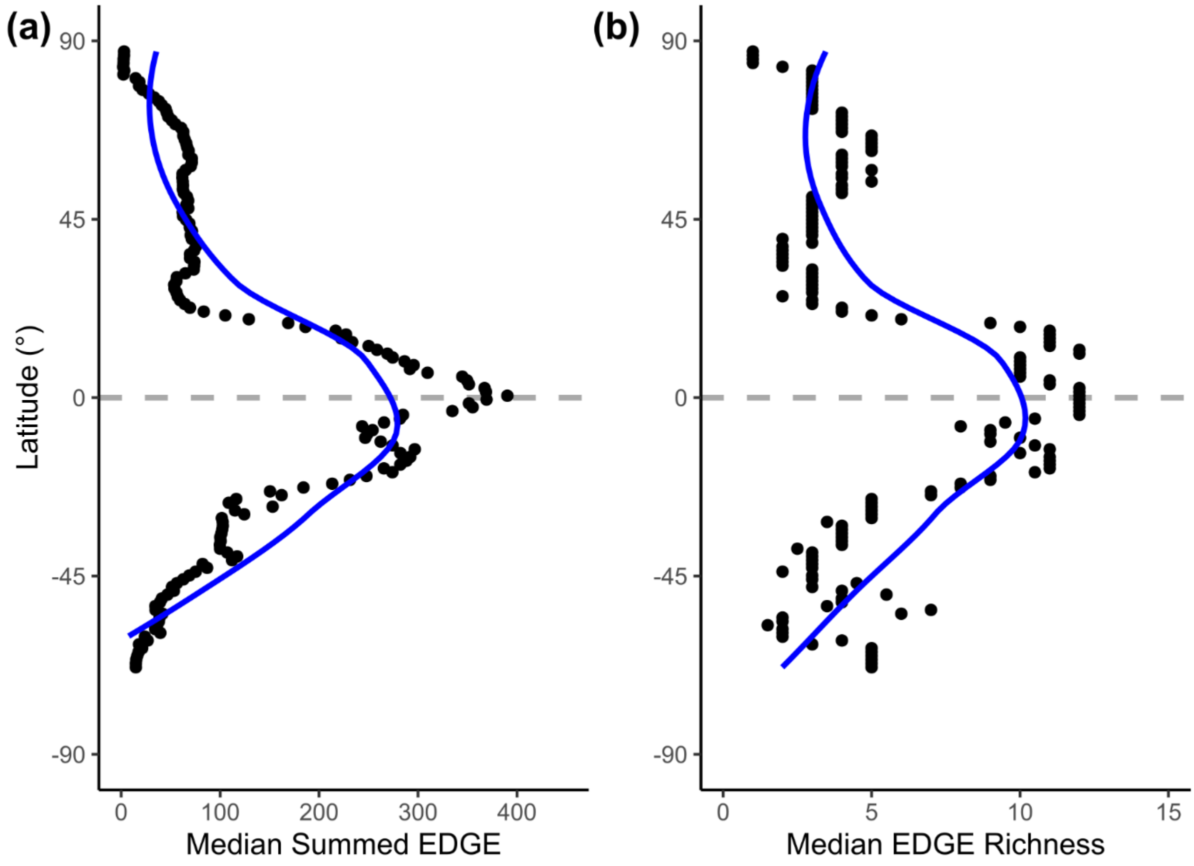

Supplementary Figure 2. Regressions of latitude against threatened evolutionary history and EDGE species richness. Loess regression (blue line) of (a) threatened evolutionary history (given by the median summed EDGE score in millions of years) and (b) the median EDGE richness at different degrees of latitude. Points reflect the median score at each degree. Source data are provided as a Source Data file.

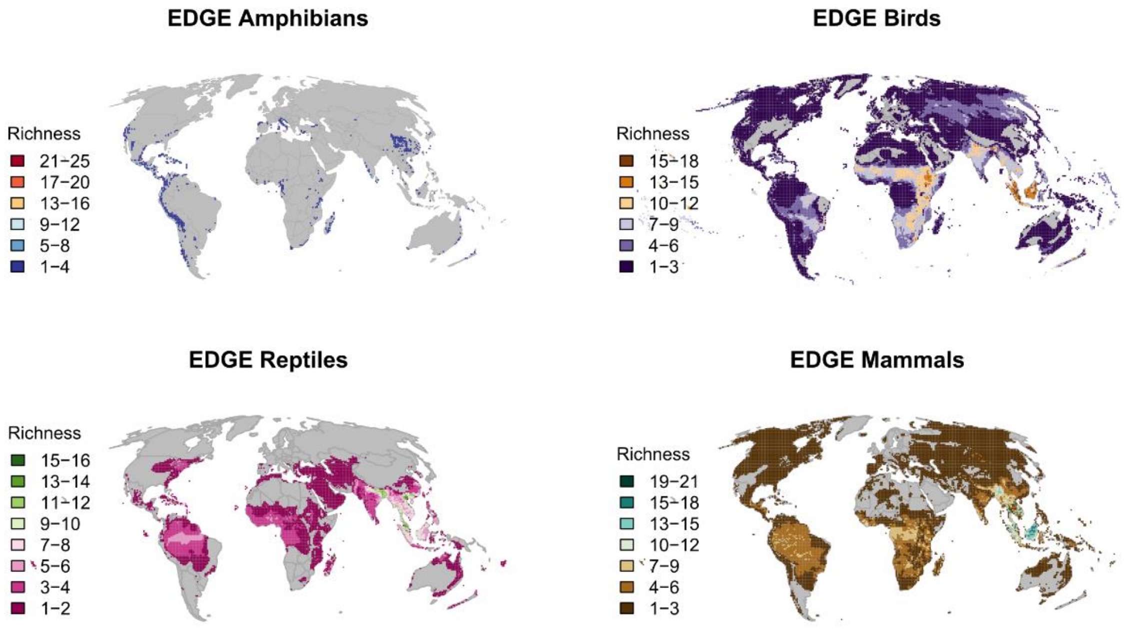

Supplementary Figure 3. EDGE tetrapod species richness. The distribution of Evolutionarily Distinct and Globally Endangered (EDGE) amphibians, birds, reptiles, and mammals**.** Source data are provided as a Source Data file.

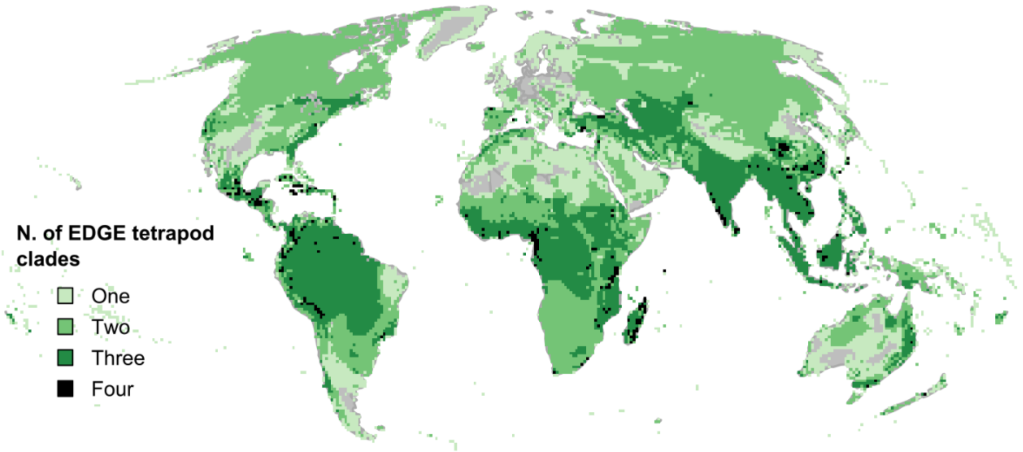

Supplementary Figure 4. Spatial congruence of EDGE tetrapod groups. The distribution co-occurrence of Evolutionarily Distinct and Globally Endangered (EDGE) species from four tetrapod groups. Source data are provided as a Source Data file.

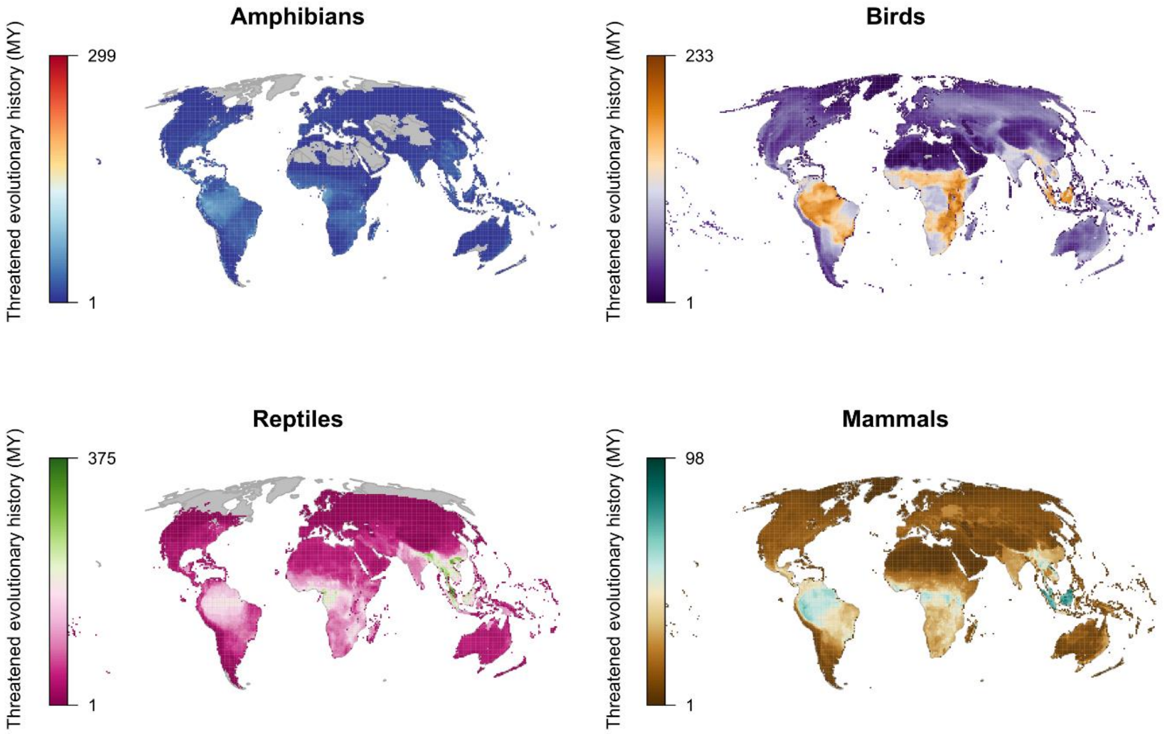

Supplementary Figure 5. Threatened evolutionary history of tetrapod groups. Threatened evolutionary history is represented in millions of years (MY) and mapped using summed EDGE scores. Source data are provided as a Source Data file.

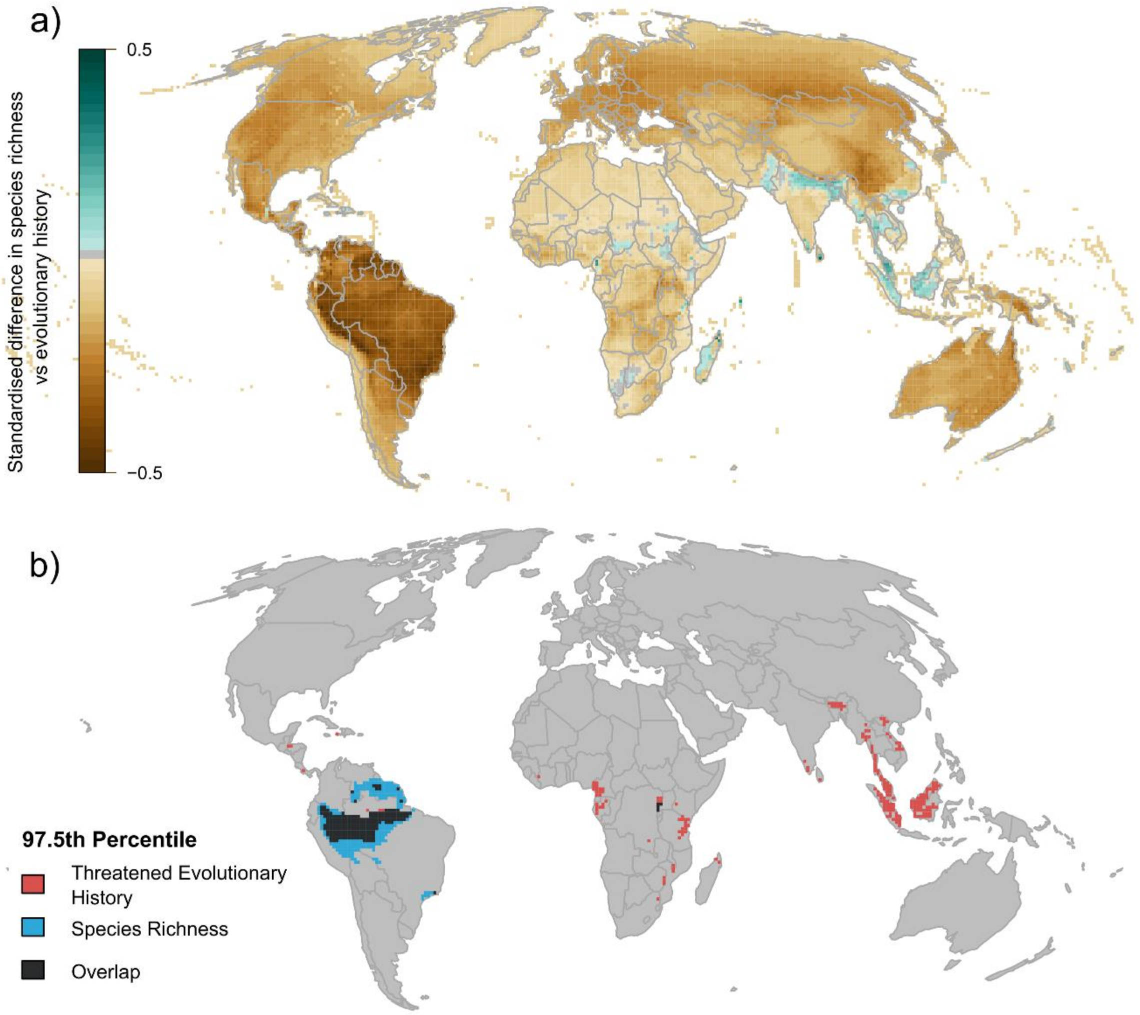

Supplementary Figure 6. Comparison of species richness and threatened evolutionary history. (a) The difference in the standardised scores of threatened evolutionary history and species richness per grid cell, where positive values represent greater standardised threatened evolutionary history (green) and negative values represent greater standardised species richness (brown). (b) The 97.5th percentile of threatened evolutionary history grid cells in red and species richness in blue, with the overlap shown in black. Threatened evolutionary history is measured using summed EDGE scores. Source data are provided as a Source Data file.

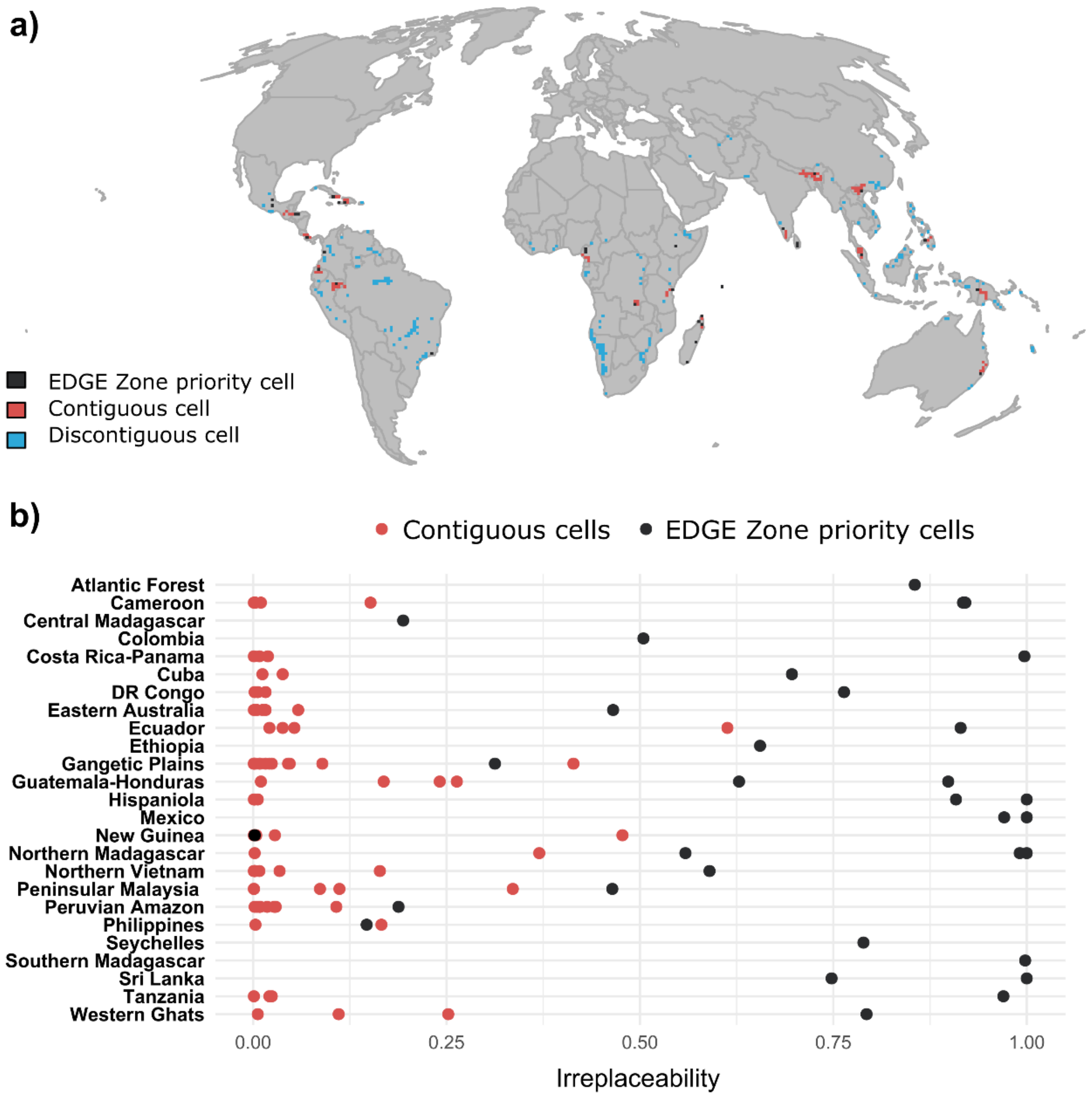

Supplementary Figure 7. The irreplaceability of EDGE Zone grid cells. The (a) location of the grid cells from the uncertainty analysis, where EDGE Zone priority cells are those identified from the complementarity procedure using median EDGE scores (black). Other highlighted cells are those identified from the complementarity procedure repeated on the distribution of 1000 possible EDGE scores, coloured coded by whether they are contiguous (red) or discontiguous (blue) to EDGE Zone priority cells. The irreplaceability of EDGE Zone cells (priority cells paired with contiguous cells) is shown in panel (b), where irreplaceability refers to the proportional frequency in which cells were selected from 1000 iterations. Source data are provided as a Source Data file.

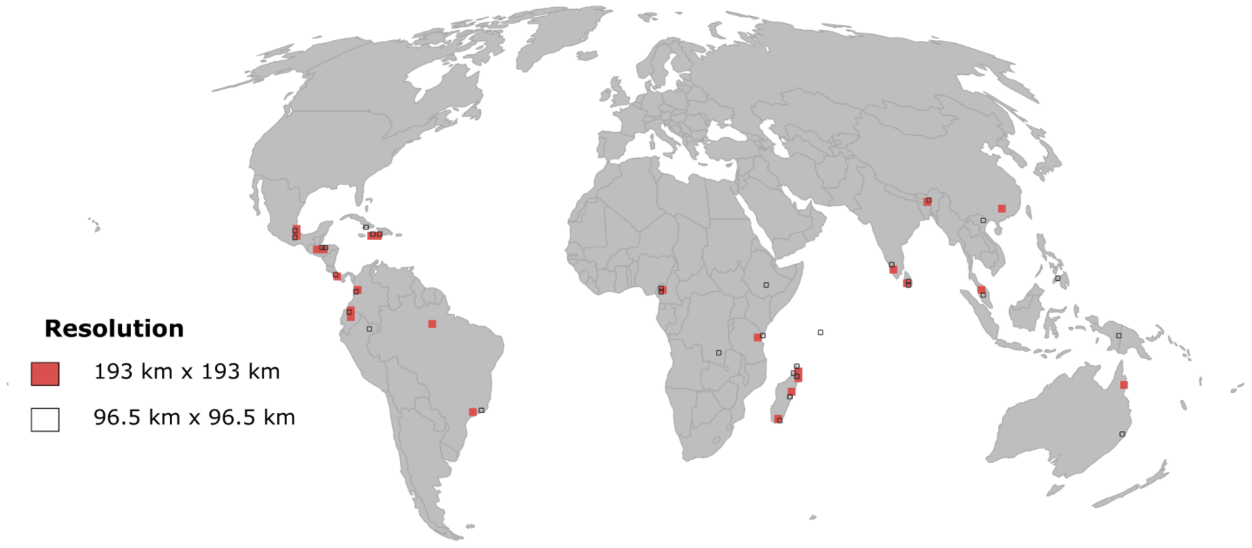

Supplementary Figure 8. Threatened evolutionary history prioritisation with complementarity at a resolution of 193 km x 193 km. Cells were selected iteratively based on those with the highest summed EDGE score using spatial complementarity at a resolution of 193 km 193 km (in red). Priority cells selected using a 96.5 km x 96.5 km resolution are shown in a black outline. Source data are provided as a Source Data file.

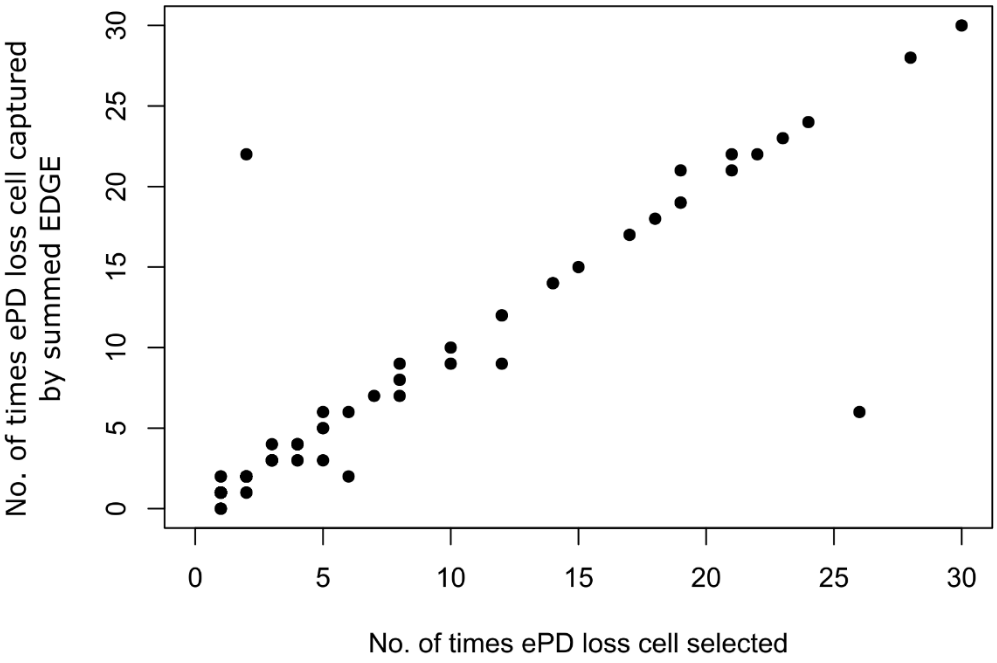

Supplementary Figure 9. Comparison of a branch-length vs a median EDGE score approach for the selection of priority grid cells. The complementarity procedure was compared across 30 iterations based on the distribution of threat-weighted phylogenetic trees used in Gumbs et al. (2022b); cells were selected based on calculations of expected phylogenetic diversity loss (ePD loss; the branch-length approach) and on species-specific EDGE scores calculated from the same trees. The plot displays the frequency in which cells selected using calculations of ePD loss were also selected using summed EDGE scores, showing a strong correlation using spearman's rank ($\rho = 0.958$, $p < 0.0001$). There was a 97.7% overlap in the location of cells selected between methods. Source data are provided as a Source Data file.

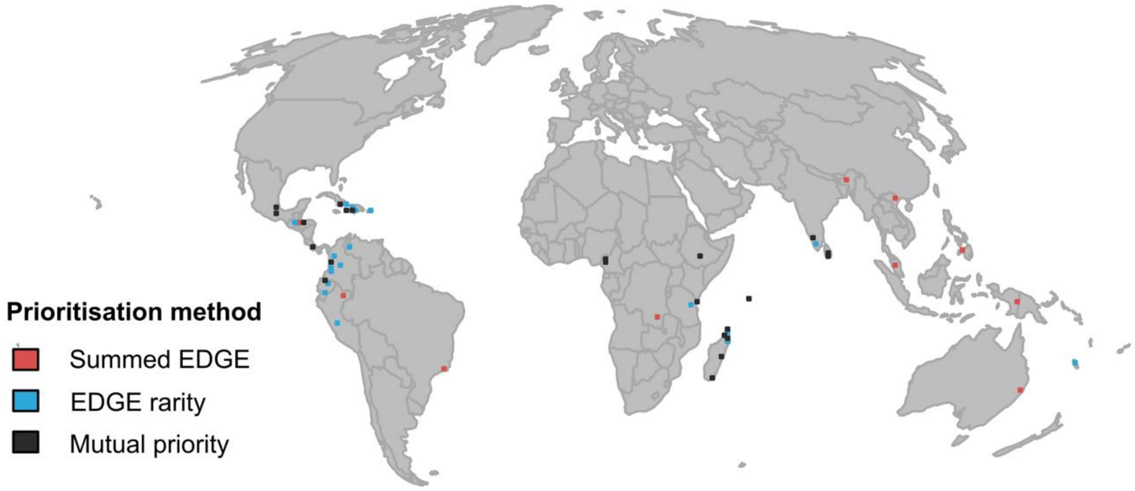

Supplementary Figure 10. EDGE rarity prioritisation with complementarity. The map shows priority grid cells based on summed EDGE scores in red and EDGE rarity scores in blue, with sites of mutual overlap shown in black. Here, we define the EDGE rarity of a species as its EDGE score divided by the number of grid cells its range overlaps with. For both metrics, priority cells were selected iteratively with complementarity until the pooled species composition represented 25% of threatened tetrapod evolutionary history. Source data are provided as a Source Data file.

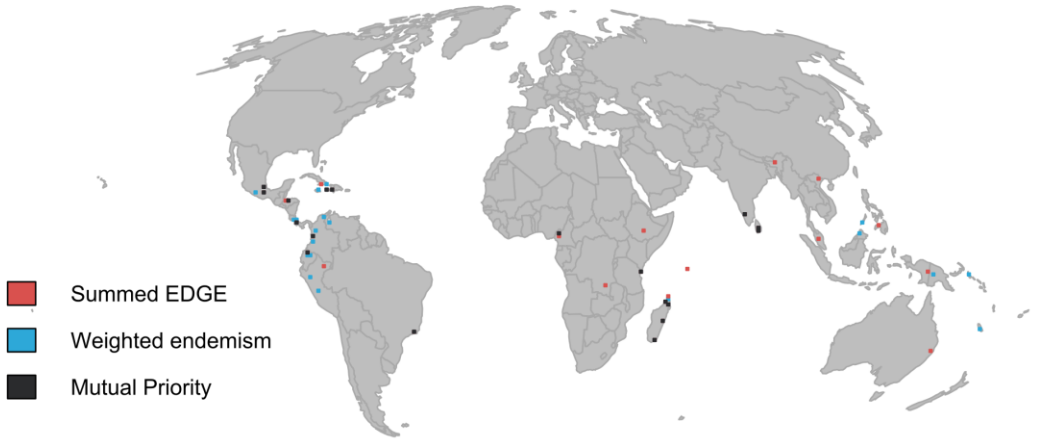

Supplementary Figure 11. Weighted endemism prioritisation. The map shows priority grid cells based on summed EDGE scores in red and weighted endemism scores in blue, with sites of mutual priority in black. Priority cells were selected iteratively with complementarity until the pooled species composition represented 25% of either threatened tetrapod evolutionary history for the summed EDGE score approach, or species richness for the weighted endemism approach. Source data are provided as a Source Data file.

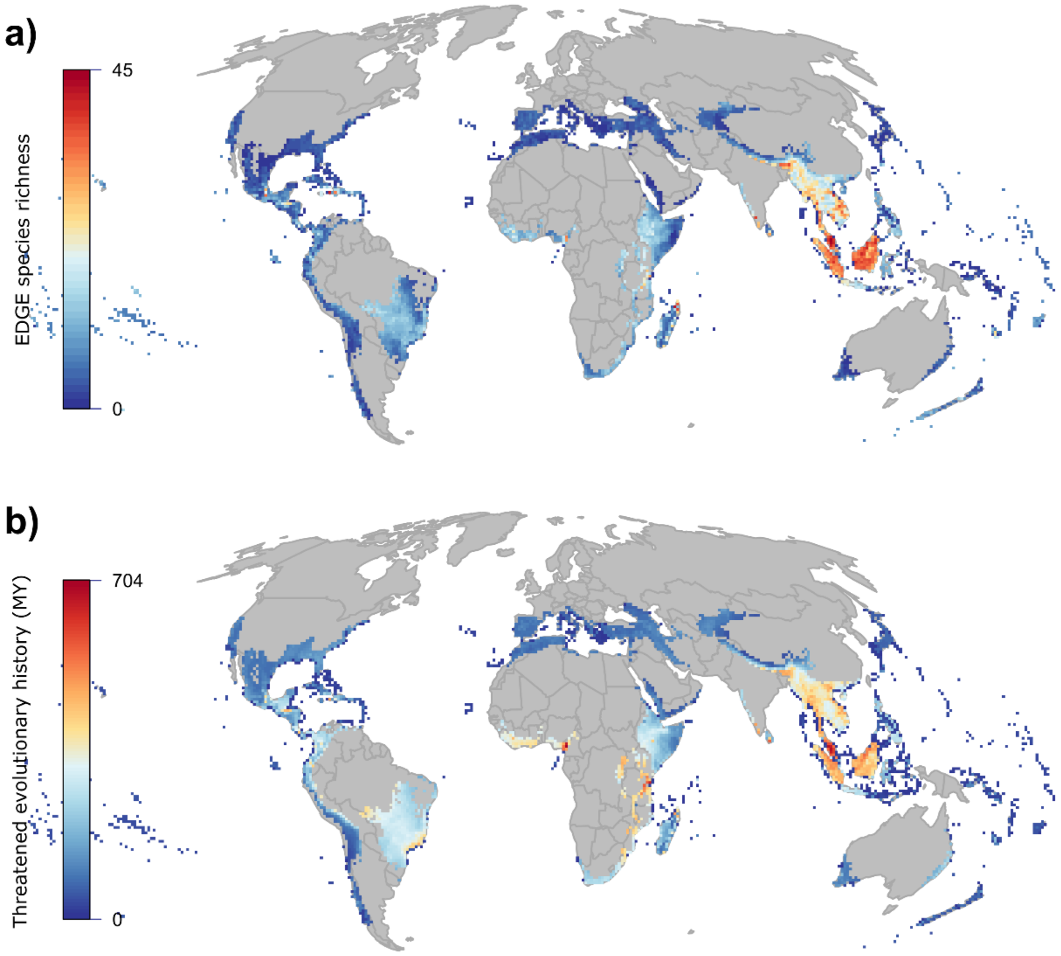

Supplementary Figure 12. Biodiversity Hotspots. Biodiversity Hotspots, as defined by Myers et al. (2000), mapped in terms of their (a) threatened evolutionary history (using summed EDGE scores, given in MY) and (b) their EDGE tetrapod species richness at 96.5 km x 96.5 km resolution. Source data are provided as a Source Data file.

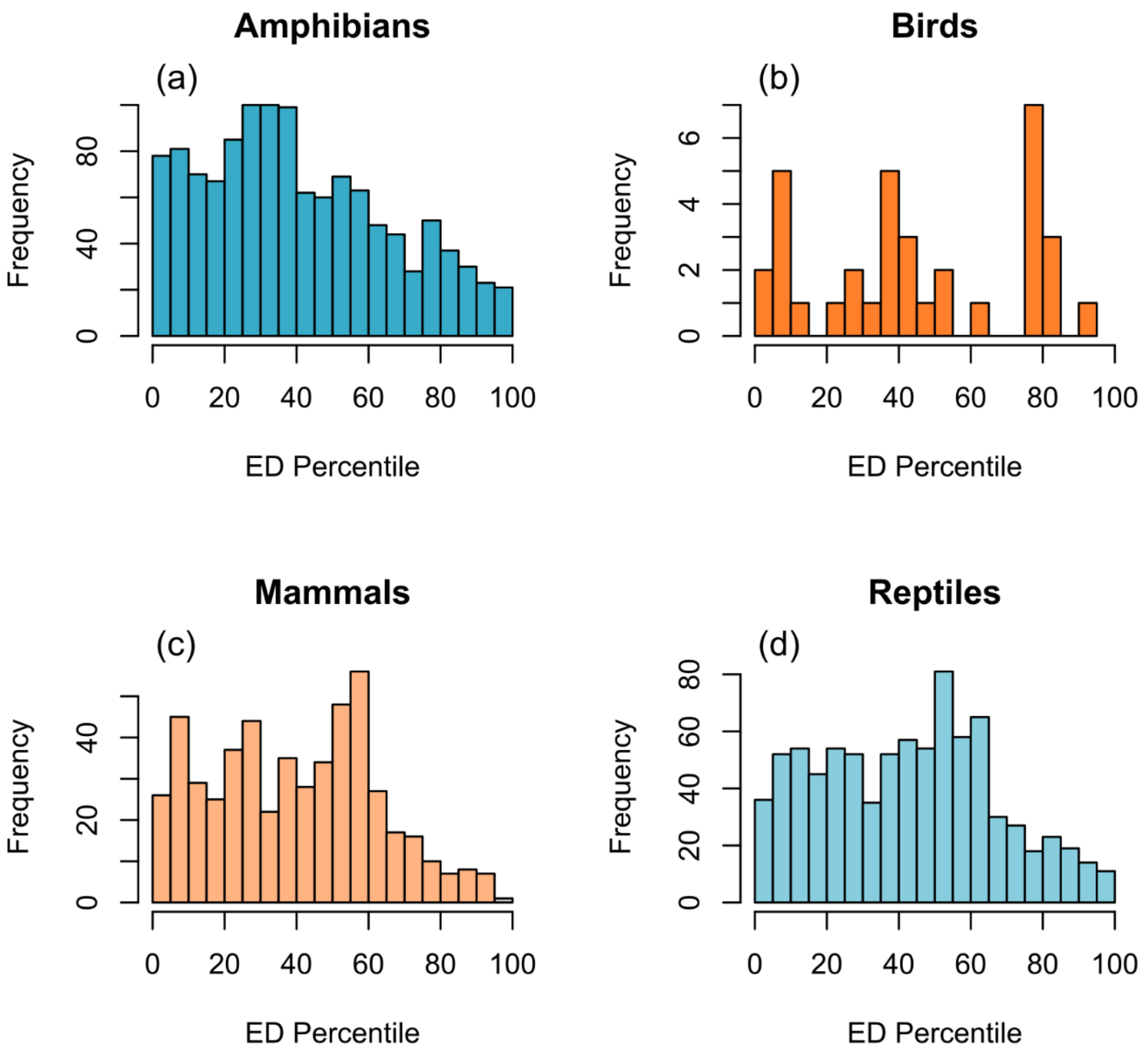

Supplementary Figure 13. Evolutionary Distinctiveness of tetrapod species with absent range data. Histograms of the Evolutionary Distinctiveness (ED) of species with absent range data visualised across 20 percentiles for (a) amphibians (Median ED = 7.32, Median Percentile = 37th, n = 1215), (b) birds (Median ED = 2.82, Median Percentile = 43rd, n = 35), (c) mammals (Median ED = 1.64, Median Percentile = 40th, n = 522), and (d) reptiles (Median ED = 5.59, Median Percentile = 44th, n = 837). Source data are provided as a Source Data file.

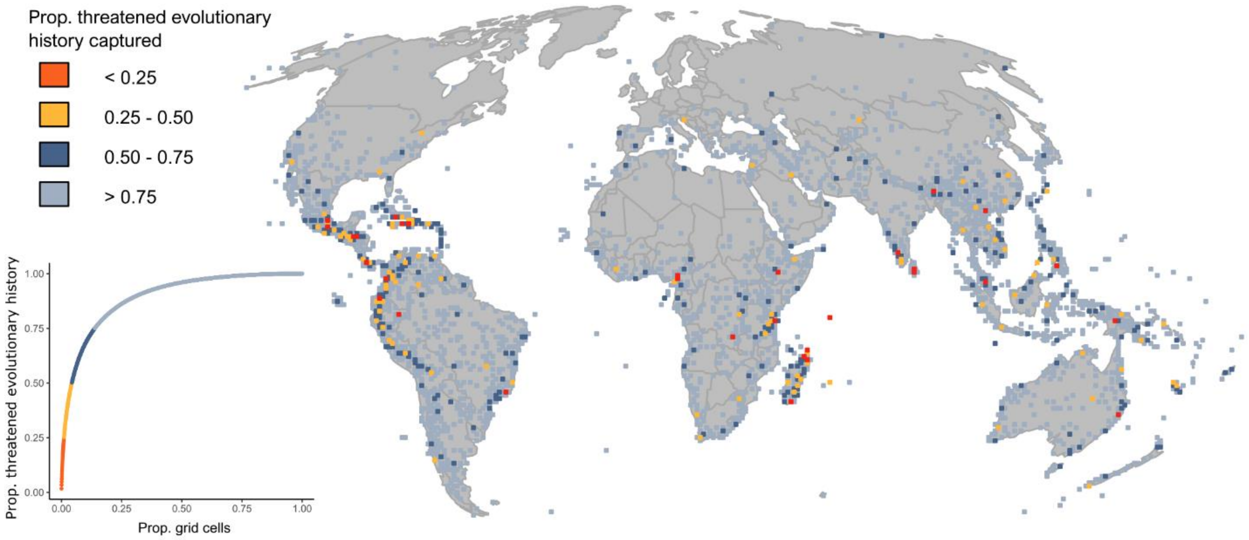

Supplementary Figure 14. The complementary set of grid cells needed to capture 100% of threatened tetrapod evolutionary history. 3001 cells, identified iteratively based on the highest scoring summed EDGE values, were selected to capture the threatened evolutionary history of all 33,628 mapped tetrapod species. Cells are coloured coded by the proportion of threatened evolutionary history captured by component cells in 25% intervals. Source data are provided as a Source Data file.

Supplementary Note 1. A comparison of using summed EDGE scores vs phylogenetic branch lengths for the calculation of threatened evolutionary history. The (a) overlap in priority sites of threatened evolutionary history calculated using summed EDGE scores vs phylogenetic branch lengths at six different percentiles and (b) the results of a Pearson's correlation adjusted for spatial autocorrelation.

A. Overlap in areas at six percentiles

	**Percentiles**					
**Tetrapod group**	**80th**	**85th**	**90th**	**95th**	**97.5th**	**99th**
**Mammals**	0.966	0.962	0.949	0.944	0.917	0.903
**Amphibians**	0.998	0.998	0.998	0.994	0.997	0.992
**Birds**	0.999	0.999	0.999	0.998	0.995	0.994
**Squamates**	0.990	0.981	0.980	0.972	0.932	0.949

B. Correlation

**Tetrapod group**	**R**	***P* value**	***F* statistic**	**DF**
**Mammals**	0.995	<0. 0001	90	37
**Amphibians**	0.998	<0. 0001	259	106
**Birds**	1.000	<0. 0001	39238	37
**Squamates**	0.999	<0. 0001	946	22

Supplementary Note 2. Predictors of irreplaceability in EDGE Zone grid cells. The effects of five variables on the irreplaceability scores of 112 EDGE Zone grid cells, assessed using General Linear Models with a binomial family distribution. Significant *p* values at the 5% criterion are bolded.

	Estimate	Std. error	*z* value	*p* value
Intercept	-2.671	1.688	-1.582	0.113
Proportion of endemic species	24.916	12.659	1.968	**0.049**
Summed EDGE score	0.008	0.002	3.091	**0.002**
Proportion of Endemic EDGE species	51.815	34.733	1.492	0.136
Median EDGE score of constituent species	-11.426	7.066	-1.617	0.106
Proportion of EDGE species	-3.223	19.185	-0.168	0.867

Supplementary Note 3. Comparison of the tetrapod threatened evolutionary history within and outside of EDGE Zones. The (a) median ED scores and (b) median EDGE scores of species from each tetrapod group stratified by whether the grid cell is found within or outside of an EDGE Zone. (c) The proportional contribution each tetrapod group made to the threatened evolutionary history in each grid cell relative to their richness. Here, a higher proportional contribution means a group contributes more to the threatened evolutionary history than expected relative to their richness. Comparisons were made using ANOVA with Tukey's Honest Significant Difference Test.

ED median:

- Within EDGE zones: Amphibian median EDE sig. greater than other groups ($p < 0.0001$ for all pairwise comparisons), reptiles greater than mammals/birds ($p < 0.0001$).
- Outside EDGE zones: Amphibian median ED sig. greater than other groups ($p < 0.0001$ for all pairwise comparisons), reptiles greater than mammals/birds ($p < 0.0001$), birds greater than mammals ($p < 0.0001$).

EDGE median:

- Within EDGE zones: Amphibian median EDGE sig. greater than other groups ($p < 0.001$ for all pairwise comparisons), reptiles greater than mammals/birds ($p < 0.0001$), no sig. difference between mammals and birds.
- Outside EDGE zones: Reptile median EDGE sig. greater than all other groups ($p < 0.0001$), amphibians greater than birds/mammals ($p <0.0001$), birds sig. greater than mammals ($p < 0.0001$).

Proportion contribution to threatened evolutionary history:

- Within EDGE zones: No difference between amphibian and reptile contributions ($p = 0.998$), both sig. greater than the other groups ($p < 0.0001$ for all pairwise comparisons), no difference between mammals and birds.
- Outside EDGE zones: Reptile contributions sig. greater than all other groups ($p < 0.0001$), amphibians greater than birds/mammals ($p <0.0001$), birds sig. greater than mammals ($p < 0.0001$).
