# Supplementary material for: Advancing EDGE Zones to identify spatial conservation priorities of tetrapod evolutionary history
Source: Nat Commun. 2024 Sep 3;15:7672. doi: 10.1038/s41467-024-51992-5 (PMC11377708; doi:10.1038/s41467-024-51992-5)
Supplement: Supplementary file 13 — Reporting Summary [file 41467_2024_51992_MOESM13_ESM.pdf]

nature portfolio

Corresponding author(s): Sebastian Pipins

Last updated by author(s): Aug 12, 2024

Reporting Summary

Nature Portfolio wishes to improve the reproducibility of the work that we publish. This form provides structure for consistency and transparency in reporting. For further information on Nature Portfolio policies, see our Editorial Policies and the Editorial Policy Checklist.

Statistics

For all statistical analyses, confirm that the following items are present in the figure legend, table legend, main text, or Methods section.

n/a	Confirmed	
☐	☒	The exact sample size (*n*) for each experimental group/condition, given as a discrete number and unit of measurement
☐	☒	A statement on whether measurements were taken from distinct samples or whether the same sample was measured repeatedly
☐	☒	The statistical test(s) used AND whether they are one- or two-sided<br>*Only common tests should be described solely by name; describe more complex techniques in the Methods section.*
☒	☐	A description of all covariates tested
☐	☒	A description of any assumptions or corrections, such as tests of normality and adjustment for multiple comparisons
☐	☒	A full description of the statistical parameters including central tendency (e.g. means) or other basic estimates (e.g. regression coefficient) AND variation (e.g. standard deviation) or associated estimates of uncertainty (e.g. confidence intervals)
☐	☒	For null hypothesis testing, the test statistic (e.g. *F*, *t*, *r*) with confidence intervals, effect sizes, degrees of freedom and *P* value noted<br>*Give P values as exact values whenever suitable.*
☒	☐	For Bayesian analysis, information on the choice of priors and Markov chain Monte Carlo settings
☒	☐	For hierarchical and complex designs, identification of the appropriate level for tests and full reporting of outcomes
☐	☒	Estimates of effect sizes (e.g. Cohen's *d*, Pearson's *r*), indicating how they were calculated

Our web collection on statistics for biologists contains articles on many of the points above.

Software and code

Policy information about availability of computer code

Data collection	All analyses took place in R version 4.1.0
Data analysis	The prioritisation code is available from https://github.com/sebpipins/EDGE-Zone-prioritisation/

For manuscripts utilizing custom algorithms or software that are central to the research but not yet described in published literature, software must be made available to editors and reviewers. We strongly encourage code deposition in a community repository (e.g. GitHub). See the Nature Portfolio guidelines for submitting code & software for further information.

Data

Policy information about availability of data

All manuscripts must include a data availability statement. This statement should provide the following information, where applicable:

- Accession codes, unique identifiers, or web links for publicly available datasets
- A description of any restrictions on data availability
- For clinical datasets or third party data, please ensure that the statement adheres to our policy

Species distribution data was obtained from the IUCN Red List of Threatened Species (Version 2021.1) for terrestrial and freshwater mammals and amphibians79 (https://www.iucnredlist.org/resources/spatial-data-download), from BirdLife International (Version 2020.1) for birds80 (https://datazone.birdlife.org/species/requestdis), and from the Global Assessment on Reptile Distributions (Version 1.5) for reptiles29 (http://www.gardinitiative.org/data.html). We used publicly available EDGE scores from Gumbs et al.45 supplementary data (https://figshare.com/s/09ab68484ba1e49cba48). The Global Multidimensional Poverty Index53 was downloaded from: https://ophi.org.uk/. The Human Footprint Index54 was downloaded from: https://doi.org/10.5061/dryad.052q5. Protected area91 data was

downloaded from: (https://www.protectedplanet.net/). Study outputs are available at: https://doi.org/10.6084/m9.figshare.25736703. Supplementary Data 1 contains diversity indices, human pressure percentages, and protected area levels within EDGE Zones. Supplementary Data 2 contains diversity indices within Biodiversity Hotspots. Supplementary Data 3 contains a list of national EDGE species richness. Supplementary Data 4 contains a list of the EDGE species found within each identified EDGE Zone. Supplementary Data 5 contains a raster layer of tetrapod summed EDGE scores. Supplementary Data 6 contains a raster layer of EDGE species richness. Supplementary Data 7 contains a shapefile for EDGE species richness by nation. Supplementary Data 8 contains a shapefile for EDGE Zones. Supplementary Data 9 contains a list of EDGE species found in each ecoregion. Supplementary Data 10 contains a sample dataset for running the EDGE Zone prioritisation code. Source Data contains all the data needed for main and supplementary figures.

Research involving human participants, their data, or biological material

Policy information about studies with human participants or human data. See also policy information about sex, gender (identity/presentation), and sexual orientation and race, ethnicity and racism.

Reporting on sex and gender	NA
Reporting on race, ethnicity, or other socially relevant groupings	NA
Population characteristics	NA
Recruitment	NA
Ethics oversight	NA

Note that full information on the approval of the study protocol must also be provided in the manuscript.

Field-specific reporting

Please select the one below that is the best fit for your research. If you are not sure, read the appropriate sections before making your selection.

☐ Life sciences ☐ Behavioural & social sciences ☒ Ecological, evolutionary & environmental sciences

For a reference copy of the document with all sections, see nature.com/documents/nr-reporting-summary-flat.pdf

Ecological, evolutionary & environmental sciences study design

All studies must disclose on these points even when the disclosure is negative.

Study description	Threatened evolutionary history and patterns of EDGE species were mapped for all tetrapod groups. Priority regions were then selected using a spatial complementarity procedure with uncertainty incorporated.
Research sample	All described species of amphibians, birds, mammals, and reptiles with EDGE and spatial data were assessed.
Sampling strategy	All described species of amphibians, birds, mammals, and reptiles with EDGE and spatial data were assessed.
Data collection	Data was collected from published and publicly available datasets.
Timing and spatial scale	Global datasets were used, downloaded between 2021-2022.
Data exclusions	Species without spatial data were excluded from the mapping analyses.
Reproducibility	The prioritisation was repeated 1000 times using a distribution of EDGE scores to account for phylogenetic and extinction-risk uncertainity.
Randomization	NA
Blinding	NA

Did the study involve field work? ☐ Yes ☒ No

Reporting for specific materials, systems and methods

We require information from authors about some types of materials, experimental systems and methods used in many studies. Here, indicate whether each material, system or method listed is relevant to your study. If you are not sure if a list item applies to your research, read the appropriate section before selecting a response.

Materials & experimental systems

n/a	Involved in the study
☒	☐ Antibodies
☒	☐ Eukaryotic cell lines
☒	☐ Palaeontology and archaeology
☒	☐ Animals and other organisms
☒	☐ Clinical data
☒	☐ Dual use research of concern
☒	☐ Plants

Methods

n/a	Involved in the study
☒	☐ ChIP-seq
☒	☐ Flow cytometry
☒	☐ MRI-based neuroimaging

Plants

Seed stocks

Report on the source of all seed stocks or other plant material used. If applicable, state the seed stock centre and catalogue number. If plant specimens were collected from the field, describe the collection location, date and sampling procedures.

Novel plant genotypes

Describe the methods by which all novel plant genotypes were produced. This includes those generated by transgenic approaches, gene editing, chemical/radiation-based mutagenesis and hybridization. For transgenic lines, describe the transformation method, the number of independent lines analyzed and the generation upon which experiments were performed. For gene-edited lines, describe the editor used, the endogenous sequence targeted for editing, the targeting guide RNA sequence (if applicable) and how the editor was applied.

Authentication

Describe any authentication procedures for each seed stock used or novel genotype generated. Describe any experiments used to assess the effect of a mutation and, where applicable, how potential secondary effects (e.g. second site T-DNA insertions, mosiacism, off-target gene editing) were examined.
